# Complete chloroplast genome sequence of *Semiliquidambar cathayensis* (hamamelidaceae), a rare and endangered species endemic to China

**DOI:** 10.1080/23802359.2019.1670114

**Published:** 2019-09-25

**Authors:** Yancai Shi, Na Duan, Bingbing Liu

**Affiliations:** a Guangxi Institute of Botany, Guangxi Zhuang Autonomous Region and Chinese Academy of Sciences, Guilin, China;; b Institute of Loess Plateau, Shanxi University, Taiyuan, China

**Keywords:** Semiliquidambar, chloroplast genome, phylogenetic analysis

## Abstract

*Semiliquidambar cathayensis* (Hamamelidaceae) is a rare and endangered species endemic to China. Here, we first report and characterize the complete chloroplast genome sequence of *S. cathayensis* based on Illumina paired-end sequencing data. The complete plastid genome was 160,406 bp in length, which contained two inverted repeats (IRs) of 26,282 bp separated by a large single-copy (LSC) and a small single copy (SSC) of 88,920 bp and 18,922 bp, respectively. The cpDNA contains 132 genes, comprising 86 protein-coding genes, 37 tRNA genes, 8 rRNA genes and one processed pseudogene. The overall GC content of the plastome is 37.9%. The phylogenetic analysis of 20 selected chloroplast genomes demonstrated that *S. cathayensis* was close to the species *Liquidambar formosana*.

*Semiliquidambar cathayensis* Hung T. Chang, an evergreen tree which belongs to the family Hamamelidaceae, is a rare and endangered species endemic to China. It’s mainly distributed in the southern regions of China, such as Guangxi and Fujian. *S. cathayensis* plays an important role in traditional Chinese medicine, owing to its anti-inflammatory activities (Zhuang et al. 2019). Furthermore, it’s also a fine landscape trees and valuable timber (Zhao et al. 2010). Due to its vulnerability to environmental changes, *S. cathayensis* is treated as rare and endangered species in China and has been registered on the China Species Red List (Fu 1991). It is thus urgent to take effective measures to conserve this vulnerable and rare species. Herein, we first report and characterize the complete plastome of *S. cathayensis* based on Illumina paired-end sequencing data, which will contribute to the further studies on its genetic research and resource utilization. The annotated cp genome of *S. cathayensis* has been deposited into GenBank with the accession number MN410884.

In this study, *S. cathayensis* was sampled from in Guangxi Zhuang Autonomous Region of China, located at 110°0′41″E, 25°47′53″N. A voucher specimen (Y.-C. Shi et al. H026) was deposited in the Guangxi Key Laboratory of Plant Conservation and Restoration Ecology in Karst Terrain, Guangxi Institute of Botany, Guangxi Zhuang Autonomous Region and Chinese Academy of Sciences, Guilin, China. The experiment procedure is as reported in Zhang et al. (2019). Around 2 Gb clean data were used for the cp genome de novo assembly by the program NOVOPlasty (Dierckxsens et al. 2017) and direct-viewing in Geneious R11 (Biomatters Ltd., Auckland, New Zealand). Annotation was performed with the program Plann (Huang and Cronk 2015) and Sequin (http://www.ncbi.nlm.nih.gov/).

The chloroplast genome of *S. cathayensis* is a typical quadripartite structure with a length of 160,406 bp, which contained inverted repeats (IR) of 26,282 bp separated by a large single-copy (LSC) and a small single copy (SSC) of 88,920 bp and 18,922 bp, respectively. The cpDNA contains 132 genes, comprising 86 protein-coding genes, 37 tRNA genes, 8 rRNA genes and one processed pseudogene. Among the annotated genes, 15 of them contain one intron (*atp*F, *ndh*A, *ndh*B, *rps*16, *rpoC*1, *pet*B, *pet*D, *rpl*16, *rpl*2, *trn*A-UGC, *trn*I-GAU, *trn*G-GCC, *trn*K-UUU, *trn*L-UAA and *trn*V-UAC), and three genes (*clp*P, *rps*12 and *ycf*3) contain two introns. The overall GC content of the plastome is 37.9%.

To identify the phylogenetic position of *S. cathayensis*, phylogenetic analysis was conducted. A neighbor joining (NJ) tree with 1000 bootstrap replicates was inferred using MEGA version 7 (Kumar et al. 2016) from alignments created by the MAFFT (Katoh and Standley 2013) using plastid genomes of 17 species. It showed the position of *H. sericophylla* was close to the species *Liquidambar formosana* (Figure 1). Our findings can be further used for population genomic and phylogenomic studies of Hamamelidaceae. It will also provide fundamental data for the conservation, utilization and management of this rare species.

**Figure 1.**
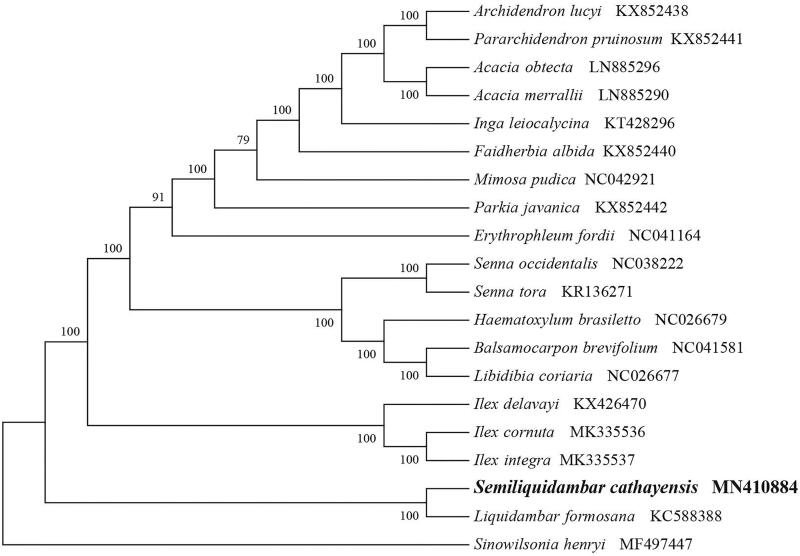
NJ phylogenetic tree of S. cathayensis with 19 species was constructed by chloroplast plastome sequences. Numbers on the nodes are bootstrap values from 1000 replicates. Sinowilsonia henryi was selected as outgroups.
